# SNPs in folate pathway are associated with the risk of nonsyndromic cleft lip with or without cleft palate, a meta-analysis

**DOI:** 10.1042/BSR20194261

**Published:** 2020-03-18

**Authors:** Qiuyan Li, Lidan Xu, Xueyuan Jia, Komal Saleem, Tahir Zaib, Wenjing Sun, Songbin Fu

**Affiliations:** 1 Laboratory of Medical Genetics, Harbin Medical University, Harbin, China; 2 Key Laboratory of Preservation of Human Genetic Resources and Disease Control in China (Harbin Medical University), Ministry of Education, China; 3 Editorial Department of International Journal of Genetics, Harbin Medical University, Harbin, China

**Keywords:** Cleft Lip, Folate, MTHFR, MTRR, SNP

## Abstract

**Background:** Prenatal intake of folic acid is important for prevention of NSCL/P (nonsyndromic cleft lip with or without cleft palate). Associated genes in folate pathway are major enzymes of folic acid metabolism that is crucial for preventing birth defects. The present meta-analysis aims to investigate the association between four SNPs in folate pathway genes and the risk of NSCL/P.

**Methods:** Comprehensive bioinformatics analysis was used to predict the functional pathogenicity of genetic variation. The PubMed, Embase database and Google Scholar were searched by two researchers. Stata 11.0 software was used to analyze the results. Subgroup analysis was carried out to assess the influence of genetic background. Sensitivity analysis, regression analysis and publication analysis were also conducted to enhance the strength of our results.

**Results:** It is estimated that the probability of two missense mutation rs1801133 in *MTHFR* and rs1801394 in *MTRR* are more likely to be damaging by bioinformatics analysis. A significant association between rs1801133 and risk of NSCL/P in two genetic models: TT genotype vs CC genotype (OR = 1.333 95%CI = 1.062–1.674, *P* = 0.013), and recessive model (OR = 1.325 95%CI = 1.075–1.634, *P* = 0.008). A significant protective association between rs1801394 GG genotype and NSCL/P in Asian (GG vs AA, OR = 0.520 95%CI = 0.321–0.841, *P* = 0.008) was observed. Meta-regression, sensitivity analysis, and publication bias analysis confirmed that the results of the present study were statistically significant.

**Conclusions:** The present study identified that rs1801133 in *MTHFR* is associated with the risk of NSCL/P, and rs1801394 GG genotype in *MTRR* play a protective role in Asian*.* Further, larger studies should be performed to confirm these findings.

## Background

NSCL/P (Nonsyndromic cleft lip with or without cleft palate) is one of the most common birth defects, characterized by craniofacial abnormality due to incomplete separation between the nasal and oral cavities [1]. NSCL/P can influence the quality of life by affecting communication problems and contributing to dysphagia. Cleft lip and palate occur in approximately one in 500–700 live births worldwide. Cleft lip is a hereditary disease with polygenic inheritance, however, the underlying genetic cause and fundamental molecular mechanism of the disease remains still elusive. However, the incidence of cleft lip varies substantially across different ethnic groups and geographical areas (http://www.who.int/oral_health/publications/factsheet/en/).

**Figure 1:**
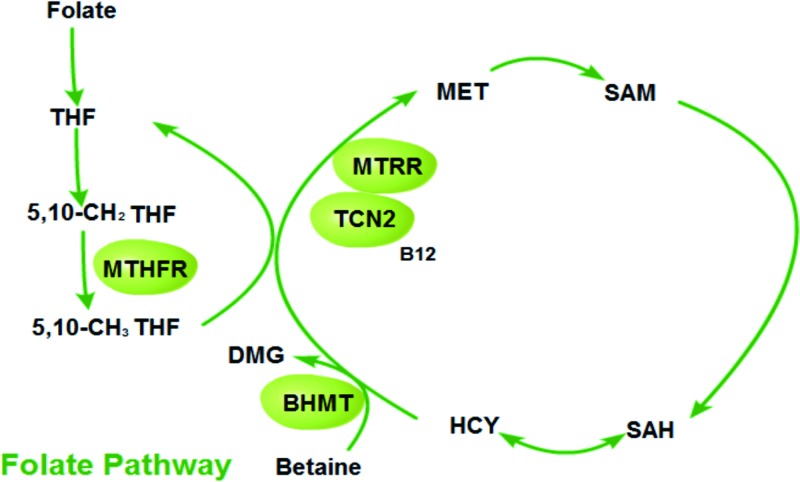
Folate pathway Abbreviations: SAH, S-adenosylhomocysteine; SAM, S-adenosyl methionine;

Although folic acid and multivitamin supplementation in prescribed period of pregnancy has been indicated as an effective method to prevent the risk of oral facial cleft. The significance of genetic locus in folate pathway and folate metabolism involved in disease pathogenesis is not clear [2,3]. Recently, many efforts have been made to find the genetic variants in folate pathway genes such as *MTHFR* (methylenetetrahydrofolate reductase), *MTRR* (Methionine synthase reductase), *TCN2* (transcobalamin 2), and *BHMT* (betaine-homocysteine methyltransferase) and their susceptibility to cleft lip [4–10]. MTHFR plays an important role in primary circulation of folate and catalyzing the reaction of 5,10-methylenetetrahydrofolate to 5-methyltetrahydrofolate. The substrate and metabolites are important for DNA biosynthesis, cell division and process during development. Currently, there is no targeted therapy for NSCL/P patients carried with *MTHFR* mutations, while there are some reports on other genetic diseases. In 2017, Martinez Saguer et al*.* reported successful management of hereditary angioedema during pregnancy in a patient carried with heterozygous *MTHFR* mutation [11]. Lahiri et al. reported successful conservative treatment of myocardial infarction in a teenager carried with *MTHFR* mutation [12]. Recently, Al-Eitan et al*.* also showed that *MTHFR* polymorphism was associated with treatment response in Jordanian population with epilepsy [13]. MTRR and TCN2 are essential in maintaining the levels of activated vitamin B12, and BHMT is vital for catalyzing betaine to dimethyl glycine (DMG), which are involved in remethylating Hcy (homocysteine) to Met (methionine) (Figure 1). The four genetic missense variations 677C>T in *MTHFR* (rs1801133), 66A>G in *MTRR* (rs1801394), 776C>G in *TCN2* (rs1801198), and 716 G>A in *BHMT* (rs3733890) have influence on protein function (Table 1), and have been reported to be associated with cleft lip. However, there are different conclusions regarding the influence of these SNPs in different populations [4–10,14–41].

**Table 1:** Information of four SNPs in the present study

SNP	Gene	Codon		Polyphen2		SIFT		CADD		PhyloP		LRT
			Score	Prediction	Score	Prediction	Score	Prediction	Score	Prediction	Score	Prediction
rs1801133	*MTHFR*	C677T	0.998	probability damaging	0.027	damage	25.0	damaging	9.137	conserved	0	deleterious
rs1801394	*MTRR*	A66G	1	probability damaging	0.064	tolerable	23.3	damaging	0.098	nonconserved	0	deleterious
rs1801198	*TCN2*	C776G	0.315	benign	0.09	tolerable	18.9	tolerable	0.081	nonconserved	0.027	neutral
rs3733890	*BHMT*	G716A	0.064	benign	0.218	tolerable	21.8	damaging	2.864	conserved	0.070	neutral

Here, a comprehensive bioinformatics analysis was used to predict the functional pathogenicity of genetic variation and a systematic review according to PRISMA2009 was performed to provide more precise statistical results. Our study could provide basic data for exploring effective therapeutic strategies for NSCL/P.

## Methods

### Literature search

All published studies before April 2019 were searched using the PubMed database, Embase database, and Google Scholar with the following terms: “NSCL/P”, “cleft lip”, “SNP”, “polymorphism”, “genetic”, “variant”, “MTHFR”, “MTHFR C677T”, “rs1801133”, “MTRR”, “MTRR A66G”, “rs1801394”, “TCN2”, “TCN2 C776G”, “rs1801198”, “BHMT”, “BHMT G716A”, and “rs3733890”. Relevant references of related articles were also included.

### Inclusion criteria and exclusion criteria

All studies were independently reviewed by two researchers. Studies were included in the meta-analysis if they met the following criteria: (1) original study of human participants; (2) an association study between rs1801133 and/or rs1801394 and/or rs1801198 and/or rs3733890 and NSCL/P; (3) case–control study or cohort study; (4) allele data were available; (5) the largest sample size or sufficient useful data were included in duplicate publications from the same population. Studies were excluded if they met the following criteria: (1) allele data were not available; (2) publications duplicate from the same population; and (3) review article and meta-analysis.

### Quality score assessment

The quality of research was evaluated to guarantee the strength of results and conclusions. The NOS (Newcastle–Ottawa scale) score was calculated to assess the quality of studies [42]. A maximum of nine scores, including selection, comparability and exposure items, could be awarded, <4, 4–6, and >6 indicate poor, moderate, and good quality, respectively. Any variances in comparison were decided by a third researcher.

### Data extraction

The data were extracted independently by two researchers from all included studies using an integrated and standardized form. The following information was extracted: (1) first author name; (2) publication year; (3) population ethnicity; and (4) genotype distribution.

### Computational and statistical analysis

Polyphen2, SIFT, CADD, phyloP, and LRT were used for bioinformatics prediction. The HWE (Hardy–Weinberg equilibrium) test was calculated by the chi-square test. The distribution of allelic frequencies in controls were considered to deviate from HWE when *P* < 0.05. STATA (11.0; Stata Corporation, College Station, TX, U.S.A.) software was used to calculate the results of meta-analysis. Heterogeneity across individual studies was assessed by Cochran's *Q* test and *I^2^* statistic (*P* < 0.10 and *I^2^* > 50% indicated evidence of heterogeneity).The fixed-effects model (Mantel–Haenszel method) was used to estimate the pooled OR when there was no evidence of the heterogeneity; otherwise, the random-effects model analyzed by DerSimonian and Laird method was used. Using rs1801133 C>T as an example: (1) allele model, T allele vs. C allele; (2) dominant model, (CT+TT vs. CC); (3) recessive model, (TT vs. CT+CC); and (4) genotype model, (CT vs. CC; TT vs. CC). The same genetic models were performed for “rs1801394”, “rs1801198”, and “rs3733890”. A *P* value of *P* < 0.05 was established as the significant difference. Two subgroups, including Caucasian and Asian, based on ethnicity were analyzed to reduce the heterogeneity and influences from the genetic background. Meta-regression, and one-way sensitivity analysis, and Egger’s regression test were also performed [43]. The trim and fill method was used when publication bias exists.

## Results

### Study characteristics

**Figure 2:**
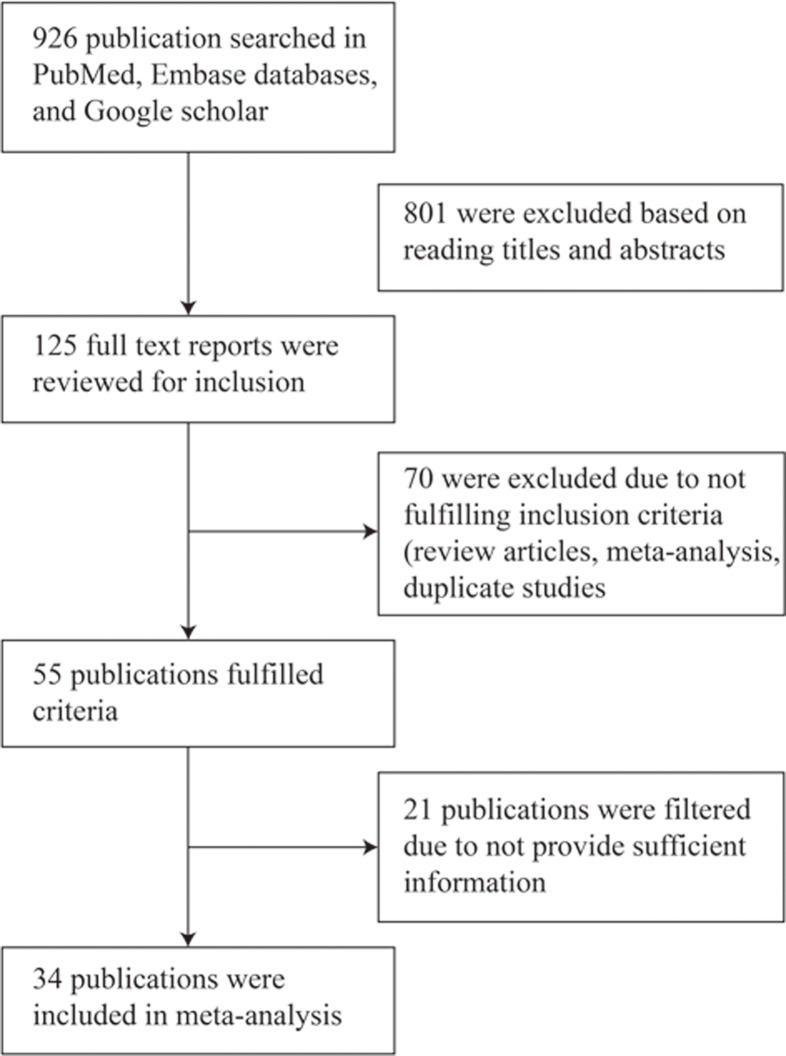
Study flow diagram

According to the search strategy, 926 publications were identified in the initial search. After evaluating the titles and abstracts, 801 publications were excluded, and 125 full-text publications were further reviewed (Figure 2). By applying the inclusion criteria, 34 publications were used for the final meta-analysis. Overall, 30 publications with 5517 cases and 7770 controls were included in the rs1801133 group; ten publications with 1767 cases and 2029 controls were included in the rs1801394 group; six publications with 1815 cases and 898 controls were included in the rs1801198 and five studies with 1253 cases and 1562 controls were included in the rs3733890 group. A total of seven studies in the control group (not excluded) were found to deviate from HWE. The main characteristics of the included publications are shown in Table 2.

**Table 2:** Characteristics of included studies about associations between four SNPs of folate pathway gene and NSCL/P

Study	Year	Ethnicity	Genotype in case					Genotype in control					*P* value of HWE test	NOS score	Source of control	Genotyping method
*MTHFR* rs1801133			Total	CC	CT	TT	AF	Total	CC	CT	TT	AF				
Shaw et al.	1998	Caucasian	310	143	127	40	0.334	383	156	178	49	0.360	0.873	6	PB	PCR-
Tolarova et al.	1998	Caucasian	111	43	49	19	0.392	106	46	52	8	0.321	0.195	6	NA	NA
Gaspar et al.	1999	Caucasian	77	30	39	8	0.357	103	49	49	5	0.286	0.096	6	HB	NA
Wyszynski et al.	2000	Caucasian	259	114	109	36	0.349	327	129	154	44	0.370	0.854	6	PB	Q-PCR, Taqman
Martinelli et al.	2001	Caucasian	64	22	30	12	0.422	106	46	43	17	0.363	0.205	6	PB	PCR
Grunert et al.	2002	Caucasian	66	34	26	6	0.288	184	90	69	25	0.323	0.052	6	PB	PCR
Shotelersuk et al.	2003	Asian	109	84	25	0	0.115	202	154	46	2	0.124	0.478	6	PB	PCR
van Rooij et al.	2003	Caucasian	105	54	45	6	0.271	128	70	54	4	0.242	0.091	6	PB	PCR
Gaspar et al.	2004	Caucasian	644	327	269	48	0.283	424	213	172	39	0.295	0.616	6	HB	PCR
Pezzetti et al.	2004	Caucasian	110	28	58	24	0.482	289	95	151	43	0.410	0.174	6	HB	PCR
Brandalize et al.	2007	Caucasian	114	49	46	19	0.368	100	45	41	14	0.345	0.353	6	HB	PCR
Chevrier et al.	2007	Caucasian	148	66	60	22	0.351	165	51	81	33	0.445	0.935	6	HB	PCR
Little et al.	2008	Caucasian	96	39	47	10	0.349	224	94	101	29	0.355	0.819	6	PB	MS-PCR
Mills et al.	2008	Caucasian	492	217	221	54	0.334	1599	715	721	163	0.327	0.341	7	HB	PCR
Ali et al.	2009	Asian	323	225	87	11	0.169	214	176	36	2	0.093	0.916	6	PB	PCR
Sozen et al.	2009	Caucasian	179	81	80	18	0.324	138	66	65	7	0.286	0.073	6	PB	PCR
Mostowska et al.	2010	Caucasian	163	81	65	17	0.304	171	78	77	16	0.319	0.629	6	PB	PCR
Ebadifar et al.	2010	Asian	61	21	18	22	0.508	215	114	72	29	0.302	0.003	6	PB	PCR
Han et al.	2011	Asian	187	46	106	35	0.471	213	74	110	29	0.394	0.236	6	HB	PCR
Aslar et al.	2013	Caucasian	80	13	57	10	0.481	125	59	62	4	0.280	0.010	6	PB	PCR
Kumari et al.	2013	Asian	467	327	125	15	0.166	469	364	100	5	0.117	0.518	6	Mixed	PCR
Murthy et al.	2014	Asian	123	104	19	0	0.077	141	107	31	3	0.131	0.672	6	HB	PCR
Estandia-Ortega et al.	2014	Caucasian	132	39	55	38	0.496	370	143	172	55	0.381	0.780	7	PB	PCR
Jiang et al.	2015	Asian	204	59	107	38	0.449	226	62	108	56	0.487	0.512	6	PB	Sequenom
Bezerra et al.	2015	Caucasian	140	74	54	12	0.279	175	85	70	20	0.314	0.341	6	PB	PCR
Abdollahi-Fakhim et al.	2015	Asian	121	38	58	25	0.446	103	27	54	22	0.476	0.605	6	PB	PCR
Wang et al.	2016	Asian	147	28	66	53	0.585	129	19	97	13	0.477	<0.001	5	PB	PCR
Marini et al.	2016	Caucasian	330	119	159	52	0.398	360	148	154	58	0.375	0.097	7	HB	Taqman
Karas Kuzelicki et al.	2018	Caucasian	103	45	45	13	0.345	199	85	96	18	0.332	0.214	7	Mixed	Taqman
Rafik et al.	2019	Africa	52	44	8	0	0.077	182	97	74	11	0.264	0.526	6	PB	PCR
*MTRR* rs1801394		Total	AA	AG	GG	AF	Total	AA	AG	GG	AF					
Brandalize et al.	2007	Caucasian	114	36	69	9	0.382	100	33	61	6	0.365	0.002	6	HB	PCR
Mostowska et al.	2010	Caucasian	164	31	81	52	0.564	166	34	70	62	0.584	0.089	6	PB	PCR
Aslar et al.	2014	Caucasian	100	14	72	14	0.500	125	13	107	5	0.468	<0.001	6	PB	PCR
Waltrick-Zambuzzi et al.	2015	Caucasian	342	95	194	53	0.439	401	136	193	72	0.420	0.806	7	HB	Q-PCR
Jiang et al.	2015	Asian	204	123	71	10	0.223	226	124	84	18	0.265	0.480	6	PB	Sequenom
Bezerra et al.	2015	Caucasian	140	98	37	5	0.168	175	112	60	3	0.189	0.111	6	PB	PCR
Murthy et al.	2015	Asian	123	42	81	0	0.329	141	65	76	0	0.270	<0.001	5	HB	PCR
Wang et al.	2016	Asian	147	71	26	50	0.429	129	29	59	41	0.547	0.380	5	PB	PCR
Marini et al.	2016	Caucasian	330	160	134	36	0.312	367	175	161	31	0.304	0.478	7	HB	Sequenom
Karas Kuzelicki et al.	2018	Caucasian	103	14	56	33	0.592	199	30	111	58	0.570	0.051	7	Mixed	PCR
*TCN 2* rs1801198		Total	CC	CG	GG	AF	Total	CC	CG	GG	AF					
Martinelli et al.	2006	Caucasian	218	85	110	23	0.358	289	89	150	50	0.433	0.330	5	PB	PCR
Mills et al.	2008	Caucasian	316	99	153	64	0.445	1097	347	532	218	0.441	0.243	7	HB	Taqman
Mostowska et al.	2010	Caucasian	163	46	88	29	0.448	181	48	103	30	0.450	0.044	6	HB	Sequenom
Jin et al.	2015	Asian	429	76	215	138	0.572	461	75	231	155	0.587	0.475	5	PB	Sequenom
Waltrick-Zambuzzi et al.	2015	Caucasian	359	139	160	60	0.390	440	179	199	62	0.367	0.576	7	Mixed	Sequenom
Marini et al.	2016	Caucasian	330	135	140	55	0.379	366	160	155	51	0.351	0.177	7	PB	PCR
*BHMT* rs3733890		Total	GG	GA	AA	AF	Total	GG	GA	AA	AF					
Mostowska et al.	2010	Caucasian	174	95	76	3	0.236	176	82	75	19	0.321	0.766	6	HB	PCR
Hu et al.	2011	Asian	166	90	56	20	0.289	268	130	118	20	0.295	0.334	5	HB	PCR
Jin et al.	2015	Asian	481	219	202	60	0.335	554	265	245	44	0.301	0.222	5	PB	PCR
Marini et al.	2016	Caucasian	330	140	150	40	0.348	366	156	163	47	0.351	0.665	7	PB	Sequenom
Karas Kuzelicki et al.	2018	Caucasian	102	42	51	9	0.338	198	98	84	16	0.293	0.734	7	HB	Q-PCR

Note: AF, allele frequency of minor allele; HB, hospital based; HWE, Hardy–Weinberg equilibrium; PB, population based.

### Associations between the four SNPs of folate pathway gene and NSCL/P in the overall population

**Figure 3:**
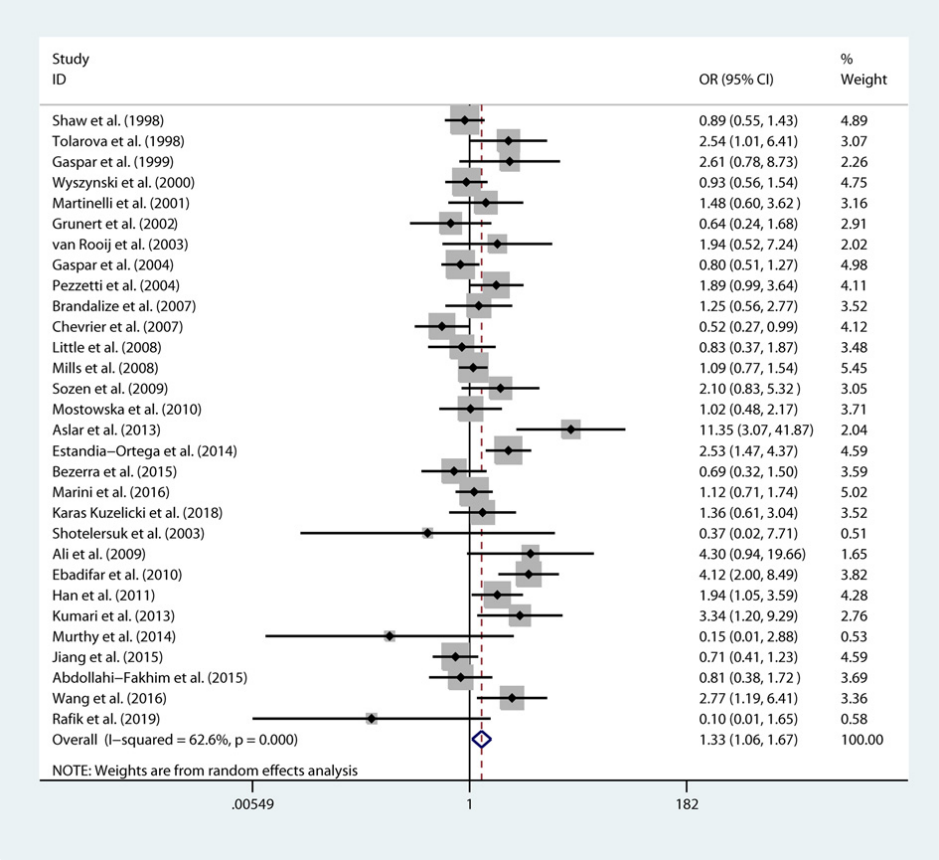
Forest plot for pooled ORs for the associations between TT vs CC model of rs1801133 and NSCL/P risk

**Figure 4:**
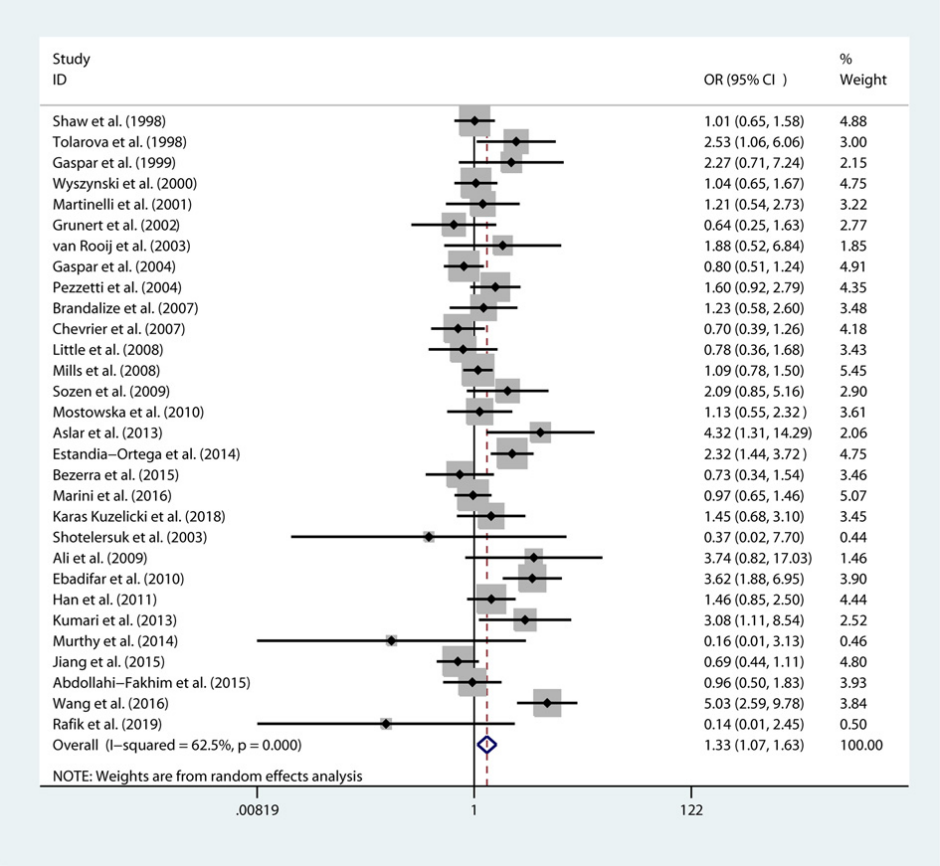
Forest plot for pooled ORs for the associations between recessive model of rs1801133 and NSCL/P risk

The meta-analysis results showed that there was a significant association between rs1801133 and NSCL/P risk in two genetic models: TT genotype vs CC genotype (OR 1.333 95% CI=1.062–1.674, *P*= 0.013) and recessive model (OR=1.325 95%CI= 1.075–1.634, *P*= 0.008) (Table 3, Figures 3 and 4). There was no statistically significant association between rs1801394 of the *MTRR*, rs1801198 of the *TCN2*, rs3733890 of the *BHMT* and NSCL/P risk in the overall population (Tables 4–6).

**Table 3:** Association between the rs1801133 (CC/CT/TT*) and NSCL/P

Genetic model	*I^2^* (%)	*P* for heterogeneity	OR (95% CI)	*P* value	*P* for publication bias	Effects model
T allele vs C allele						
Overall	73.1	0	1.111 (0.992–1.244)	0.069	0.820	random
Caucasian	58.5	0.001	1.085 (0.976–1.206)	0.131	0.361	random
Asian	79.1	0	1.244 (0.961–1.611)	0.098	0.438	random
TT vs CC						
Overall	62.6	0	1.333 (1.062–1.674)	*0.013*^#^	0.102	random
Caucasian	56.2	0.001	1.230 (0.976–1.551)	0.080	0.239	random
Asian	70.5	0.001	1.701 (0.949–3.049)	0.075	0.365	random
CT vs CC						
Overall	57.6	0	1.026 (0.901–1.169)	0.696	0.587	random
Caucasian	40.3	0.033	1.027 (0.904–1.166)	0.686	0.287	random
Asian	62.4	0.006	1.081 (0.821–1.422)	0.580	0.041	random
Dominant model						
Overall	66.2	0	1.075 (0.936–1.234)	0.305	0.804	random
Caucasian	52.8	0.003	1.067 (0.932–1.223)	0.347	0.208	random
Asian	67.9	0.002	1.172 (0.883–1.557)	0.272	0.089	random
Recessive model						
Overall	62.5	0	1.325 (1.075–1.634)	*0.008*^#^	0.220	random
Caucasian	40.9	0.030	1.190 (0.989–1.433)	0.066	0.434	random
Asian	79.1	0	1.737 (0.940–3.210)	0.078	0.404	random

Note: *wild homozygote (CC), heterozygote (CT), mutation (TT); ^#^ indicates statistically significance.

**Table 4:** Association between rs1801394 (AA/AG/GG*) and NSCL/P

Genetic model	*I^2^* (%)	*P* for heterogeneity	OR (95% CI)	*P* value	*P* for publication bias	Effects model
G allele vs A allele						
Overall	36.0	0.120	0.986 (0.895–1.085)	0.766	0.713	fixed
Caucasian	0	0.938	1.037 (0.928–1.158)	0.520	0.401	fixed
Asian	77.7	0.011	0.863 (0.569–1.309)	0.488	0.480	random
GG vs AA						
Overall	30.7	0.172	0.977 (0.781–1.223)	0.841	0.405	fixed
Caucasian	0	0.816	1.176 (0.909–1.520)	0.217	0.218	fixed
Asian	0	0.820	0.520 (0.321–0.841)	*0.008*^#^	—	fixed
AG vs AA						
Overall	78.3	0	0.879 (0.630–1.227)	0.449	0.374	random
Caucasian	31.9	0.184	1.037 (0.872–1.234)	0.679	0.443	fixed
Asian	93.2	0	0.644 (0.211–1.968)	0.441	0.599	random
Dominant model						
Overall	69.4	0.001	0.921 (0.704–1.205)	0.550	0.586	random
Caucasian	0	0.514	1.047 (0.886–1.236)	0.591	0.353	fixed
Asian	90.3	0	0.746 (0.351–1.768)	0.506	0.908	random
Recessive model						
Overall	37.8	0.117	1.032 (0.853–1.248)	0.748	0.164	fixed
Caucasian	45.1	0.090	1.062 (0.858–1.315)	0.581	0.073	fixed
Asian	39.6	0.198	0.922 (0.605–1.406)	0.707	—	fixed

Note: *wild homozygote (AA), heterozygote (AG), mutation (GG); ^#^indicates statistically significance.

**Table 5:** Association between rs1801198 (CC/CG/GG*) and NSCL/P

Genetic model	*I^2^* (%)	*P* for heterogeneity	OR (95% CI)	*P* value	*P* for publication bias	Effects model
G allele vs C allele	38.8	0.147	0.990 (0.907–1.080)	0.821	0.541	fixed
GG vs CC	43.4	0.116	0.987 (0.824–1.181)	0.883	0.438	fixed
CG vs CC	0	0.820	0.966 (0.840–1.112)	0.631	0.196	fixed
Dominant model	0	0.428	0.976 (0.855–1.114)	0.717	0.248	fixed
Recessive model	27.4	0.229	1.002 (0.860–1.167)	0.982	0.747	fixed

Note: *wild homozygote (CC), heterozygote (CG), mutation (GG).

**Table 6:** Association between rs3733890 (GG/GA/AA*) and NSCL/P

Genetic model	*I^2^* (%)	*P* for heterogeneity	OR (95% CI)	*P* value	*P* for publication bias	Effects model
A allele vs G allele	61.0	0.036	0.994 (0.820–1.204)	0.948	0.409	random
AA vs GG	73.6	0.004	0.993 (0.558–1.764)	0.980	0.182	random
GA vs GG	23.7	0.264	0.968 (0.826–1.133)	0.685	0.961	fixed
Dominant model	33.2	0.200	0.994 (0.856–1.155)	0.940	0.709	fixed
Recessive model	75.1	0.003	1.002 (0.569–1.767)	0.994	0.192	random

Note: *wild homozygote (GG), heterozygote (GA), mutation (AA).

### Subgroup analysis

To decrease the heterogeneity, and a subgroup analysis was conducted according to genetic backgroud (i) Asian and (ii) Caucasian. The results showed that there was a significant association between rs1801394 and NSCL/P risk in Asian (GG genotype vs AA genotype, OR=0.520 95% CI=0.321–0.841, *P*= 0.008), but no associations in Caucasian (Table 4), which confers a protective role of GG genotype in Asian.

### Meta-regression and influence analysis

Publication year, sample size and HWE were considered as covariates for meta-regression. The results showed that the above factors have no influence on the results (*P* >0.05). To avoid one single study affected the overall OR estimates, one-way sensitivity analysis was performed. The results showed that no study was found to exert an excessive influence on the pooled effect.

### Publication bias

There was publication bias for rs1801133 in the Asian population in genotype model CT vs CC (Table 3). Trim and fill results showed that the adjusted risk estimate unchanged, which confirmed that the results of present study are statistically reliable.

## Discussion

NSCL/P is a multifactorial disease caused by genetic and environmental factors. In previous years, various genomic susceptibility regions have been identified in association studies, linkage studies, family sequencing studies, and animal experiments suggesting that gene mutations influence the development of maxillofacial area. However, the underlying biological mechanisms remain unclear.

Folic acid is an important factor that influences the metabolism and the synthesis of nucleotides and amino acids. Previous studies have suggested that folic acid plays an important role in decreasing the risk of NSCL/P [2,3]. Folic acid metabolism is a complex process and many genes are involved in the pathway, such as *MTHFR, MTRR, TCN2*, and *BHMT*. However, there are no consistent results regarding the association between the genetic variations of these genes and NSCL/P in different populations. To clarify these inconsistent results, we carried out the meta-analysis in this study.

The present meta-analysis results demonstrated a significant association between rs1801133 and NSCL/P risk in two genetic models: TT genotype vs CC genotype (OR=1.333 95% CI=1.062–1.674, *P*= 0.013) and recessive model (OR=1.325 95%CI = 1.075–1.634, *P* = 0.008). There were a significant protective association between rs1801394 GG genotype and NSCL/P in Asian (GG genotype vs AA genotype, OR=0.520 95% CI=0.321–0.841, *P*= 0.008).

*TCN2*, encode transcobalamin2, transports vitamin B12 to cells, have been reported to be associated with multiple diseases, such as cancer, Alzheimer and other congenital abnormalities [44–46]. In 2006, Martinelli et al*.* found that the C776G in *TCN2* was associated with risk of cleft lip, but subsequent studies didn't get the significant results [10]. Similarly, the present study didn't find the significant association between the C776G and NSCL/P.

BHMT, a zinc dependent cytosolic enzyme, is important for homocysteine metabolism and methionine synthesis. In 2010, Mostowska et al*.* first found that rs3733890 of the *BHMT* was associated with NSCL/P, and other studies also indicated its association with coronary artery disease and neural tube defects [23]. In the present study, we found no evidence showing rs3733890 playing any significant role [23]. We inferred several factors may contribute to the result. First, we found a relative high value of heterogeneity among studies, which cause a different distribution of genotype. Second, the number of included studies and sample size are relatively small. So, the subgroup analysis was not conducted based on ethnicity.

MTRR plays a vital role in functional regeneration of methionine synthase, and it may be associated with increasing the congenital heart disease risk [18]. But the meta-analysis conducted by Zhang et al*.* in 2013 and Lei et al*.* in 2018 showed no association between rs1801394 and the risk of NSCL/P [47,48]. In the present study, we found a significant protective association between rs1801394 GG genotype and the NSCL/P risk in Asian, but no association in Caucasian. Considering the different background, we also summarized the data from 1000 genomes and ExAC database (S-Table 1), and we found the allelic frequencies vary in different background groups, and no significant association study between rs1801394 and the NSCL/P was found in Caucasian [6,14,25]. However, the sample size of the MTRR analysis is a limitation, and the present study did not consider the possibility of linkage disequilibrium, so further well-designed studies are required to establish these findings.

MTHFR is an important enzyme in homocysteine metabolism and C677T rs1801133 is one of the most important functional polymorphisms. Prediction by bioinformatics tools showed that the change of genetic variant will influence the protein function and predispose to cause the disease (Table 1). The allelic frequencies vary in different ethnic groups and the minor allele frequency (MAF) of *MTHFR* rs1801133 in Asian are lower than that in European and American, so it is very valuable to summarize and analyze by systematic statistical methods. In 1998, Tolarava found TT genotype of rs1801133 increase the risk of CL/P, later on, several studies also found the associations between rs1801133 and NSCL/P in different population [19,29,34]. However, there were several studies failed to find association between rs1801133 and the risk of NSCL/P [38,41]. In the present study, we included 30 studies including 5517 cases and 7770 controls and found TT genotype can increase the risk of NSCL/P.

The strength of this meta-analysis is that it expands to a large number of related studies, and the most updated publications were included. A strict procedure for search strategy, literature inclusion, data extraction, and quality assessment by two researchers was performed to guarantee the quality. Meta-regression and sensitivity analysis were also performed to strengthen the conclusions. We confirmed the previous investigation by summarizing a larger number of closely related studies.

There are some limitations in the present meta-analysis. First, studies published only in English were included in the meta-analysis, and studies published in other languages were excluded. Second, environmental factors also contribute to NSCL/P, and in the present study, non-genetic factors and other potential interactions such as age, sex, folate level were not included in the analysis due to insufficient information.

## Conclusion

In the present study, we successfully identified rs1801133 in *MTHFR* is associated with the increasing risk of NSCL/P, and GG of rs1801394 in *MTRR* confers a protective role in Asian*.* Further well-designed studies are required to establish these findings.

## Supplementary Material

Supplementary Table S1

## Data Availability

All the data in the present research is contained in this manuscript.

## Abbreviations

*BHMT*: betaine-homocysteine methyltransferase
HWE: Hardy–Weinberg equilibrium
MAF: minor allele frequency
*MTHFR*: methylenetetrahydrofolate reductase
*MTRR*: methionine synthase reductase
NSCL/P: nonsyndromic cleft lip with or without cleft palate
*TCN2*: transcobalamin
