# Supplementary material for: SNPs in folate pathway are associated with the risk of nonsyndromic cleft lip with or without cleft palate, a meta-analysis
Source: Biosci Rep. 2020 Mar 18;40(3):BSR20194261. doi: 10.1042/BSR20194261 (PMC7080646; doi:10.1042/BSR20194261)
Supplement: Supplementary Table S1 [file BSR-2019-4261_supp.pdf]

S-Table 1. Allele frequencies of four SNPs in different populations.

SNP	Gene	Codon	1000 genomes					ExAC				
			EUR	EAS	SAS	AFR	AMR	NFE	EAS	SAS	AFR	AMR
rs1801133	*MTHFR*	C677T	0.3648	0.2956	0.1186	0.09	0.4741	0.3450	0.3052	0.1409	0.1124	0.5141
rs1801394	*MTRR*	A66G	0.5229	0.2629	0.5245	0.2458	0.2810	0.5485	0.2810	0.5220	0.2773	0.2250
rs1801198	*TCN2*	C776G	0.4284	0.5556	0.5910	0.2186	0.3559	0.4297	0.5690	0.5886	0.2402	0.3329
rs3733890	*BHMT*	G716A	0.3231	0.3155	0.2883	0.2035	0.3775	0.2953	0.3212	0.2886	0.2204	0.3841

Note: EUR: European; EAS: East Asian; SAS: South Asian; AFR: African/African American; AMR: Admixed American; NFE: Non-Finnish European
